# Peer Violence in Youth Sport: Do Age, Year of Training, and Type of Sport Affect Outcomes?

**DOI:** 10.3390/sports13040127

**Published:** 2025-04-21

**Authors:** Brigita Banjac, Ivana M. Milovanovic, Radenko M. Matic, Stevo Popovic, Zeljka Bojanic, Emanuele Isidori, Patrik Drid

**Affiliations:** 1 Faculty of Sport and Physical Education, University of Novi Sad, 21000 Novi Sad, Serbia; brigita.banjac@fsfvns.edu.rs (B.B.); ivana.milovanovic@fsfvns.edu.rs (I.M.M.); radenko.matic@fsfvns.edu.rs (R.M.M.); patrik.drid@fsfvns.edu.rs (P.D.); 2 Sports Management and Sociology Lab, University of Novi Sad, 21000 Novi Sad, Serbia; 3 Faculty for Sport and Physical Education, University of Montenegro, 81400 Niksic, Montenegro; 4 K-CLUB, Korea University, Seoul 02841, Republic of Korea; 5 Faculty of Legal and Business Studies Dr. Lazar Vrkatic, 21000 Novi Sad, Serbia; zeljka.bojanic@gmail.com; 6 Department of Movement, Human and Health Sciences, University of Rome Foro Italico, 00135 Rome, Italy; labopedagogia@gmail.com; 7 Faculty of Medicine, University of Novi Sad, 21000 Novi Sad, Serbia

**Keywords:** aggression, interpersonal violence, youth, athletes, sport type, collective sport, contact sport

## Abstract

Background: In sports and physical activities, destructive behaviors such as aggression and violence are not uncommon. Although sports and physical activities have many benefits, they can also have negative consequences. This study aimed to investigate the factors that can contribute to the manifestation of aggression and interpersonal violence (IV). The variables incorporated were the athletes’ age, sports experience (training years), and type of sport. Methods: This study included *n* = 2091 youth athletes (aged between 11 and 18 years) from Serbia, with a cross-sectional study design. The data were collected through a questionnaire and analyzed using the Chi-square and Mann–Whitney U tests in IBM SPSS. Results: The athletes’ ages and the types of sports (collective and contact) in which they participated are associated with aggression and IV manifestation. In addition, physical violence tends to be more common among older athletes in collective and contact sports, while psychological violence is more prevalent among older athletes who train for five or more years and participate in collective and non-contact sports. Conclusions: Identifying some of the correlated factors in aggression and IV manifestation among youth athletes could help in the development of strategies to reduce these destructive behaviors in youth sports.

## 1. Introduction

Many children around the world are engaged in sports and physical activities, which have the potential to enrich their lives through various psychological, physical, and social benefits. For instance, sports and physical activities can improve mental health and socialization [1,2]. Additionally, sports are associated with enjoyment and fun [3], and they can be useful in reducing anxiety, stress, and aggression [4,5] in a socially acceptable way.

However, when it comes to the increased intensity, the parental pressure, the importance of winning, and the high value of awards in the training or competition atmosphere, aggressive, unfriendly, destructive, and antisocial behavior can appear [6,7,8]. The sports environment is, therefore, not free of aggressive and violent behavior [9,10]. Research by Milovanovic et al. has shown that violence and social exclusion are widespread among children in some European countries, although sport appears to be an environment in which violence and social exclusion can be prevented [11].

Aggression and violence are commonly investigated concepts in various fields, from medical to social sciences. There are numerous definitions of aggression in the literature [12,13,14,15,16]. Most of them include the words intent and causing harm [17]. In our study, we adopt the definition that aggression is the behavior of a person that has an intent to harm another person, who is motivated to not become a victim of the harm [14,15]. Although the terms aggression and violence are often used as synonyms, their meanings are slightly different. Aggression is a broader term, and while all violent acts are considered aggressive behavior, not every aggressive act leads to violence [18]. The definition of violence in social science is complex, as it can vary depending on the research discipline and the purpose of the study. According to the World Health Organization, violence is the intentional use of physical force or power, threatened or actual, against oneself, another person, or against a group or community, that either results in or has a high likelihood of resulting in injury, death, psychological harm, maldevelopment, or deprivation [19]. The categorization of aggression and violence can be direct/indirect, self-directed/interpersonal/collective, hostile/instrumental, overt/covert, etc. [14,15,19,20]. In this study, the types included physical, psychological, and sexual violence [21,22,23,24]. Therefore, aggression is a more complex and broader term, which includes violence and other subordinate terms (such as yelling, mockery, abuse, and bullying).

Entering adolescence can increase the likelihood of aggressive behavior [25]. Adolescents are exposed to various problems, and their unstable value system can be influenced by the aggression and interpersonal violence (IV) they see through the media [26]. Therefore, these unfavorable behaviors may be one of the behavioral strategies employed by adolescents [27].

Sport is not immune to aggression and IV [1,5]. Even though the first association of aggression in sports contexts belongs to hooliganism, there are other types and forms of these hostile behaviors. Therefore, the sports setting is no stranger to aggressive behaviors and, in some cases, can even be latently tolerant of violence [9]. For athletes, it means that they can face negative influences on their integrity, well-being, and health caused by non-accidental violence despite the benefits of physical activity [20,23,28]. Aggression can also have a negative effect on further participation in sports [29]. As mentioned earlier, aggression can be a continuum, ranging from minor acts to serious, harmful behaviors. Findings show that emotional harm is present in sporting culture, regardless of the sport level, and might have negative long-term consequences [29]. Studies have shown that bullying as a form of aggressive behavior can cause both short-term and long-term negative consequences [30]. So, it is important for children and youth to recognize signs of aggressive and violent behavior in the sports environment.

Various characteristics and factors could be associated with higher rates of aggression and IV. Studies have mostly explored factors such as gender, age, race, ethnicity, social status, sport type and setting, training hours, athletic level, power imbalance, and competition level [1,8,26,27,28,31]. Conway et al. conducted a systematic review in which, among the studies with athlete samples, the age of the athletes, the type of sport, and their level of competition accounted for higher scores for aggression [10]. Kreager et al. pointed out that there is a strong relationship between contact sports and violence, as wrestlers and football players are more likely to become involved in fights than men from other sports [32]. Addressing the manifestation of and the factors that could influence aggression and IV in sports contexts is necessary since athletes of all ages and levels are exposed to these behaviors [20].

The previous study confirms the presence of these destructive behaviors in sports, most often occurring after training or competitions and at sports camps, with a high prevalence in the dressing room [33]. Given the known presence of these negative behaviors, the objective of the current study was to investigate the factors that can contribute to the manifestation of aggression and IV (physical, psychological, and sexual). The variables incorporated were the athletes’ age, sports experience (training years), and type of sport.

## 2. Materials and Methods

The data were collected within the research project “Peer violence in children and youth sports” under project number 142-451-2573/2019-01, conducted by the Faculty of Sport and Physical Education, University of Novi Sad, Serbia.

The study employs a cross-sectional design, with data collection taking place from October 2019 until October 2020. Both the Association of Physical Education Teachers in Vojvodina and the Association for School Sports of the city of Novi Sad made contributions to the dissemination of the questionnaire among athletes who regularly played sports in a randomly selected sports club in the territory of the Autonomous Province of Vojvodina.

### 2.1. Participants

The total sample size involved *n* = 3000 participants. In total, 763 (25.43%) of them were excluded due to undelivered written informed consent for participation in the research. Additionally, 146 (4.87%) participants were excluded because of missing data or incomplete answers. Thus, the final study sample included *n* = 2091 children and youth from various cities, towns, suburbs, and villages in Vojvodina, Serbia. Their ages ranged from 11 to 18. They attended primary or high school and were active one of the following sports groups: archery, 0.1%; athletics, 1.5%; body shaping sports, 0.1% (cardio workout, CrossFit, aerobic, fitness); combat sports, 8.6% (taekwondo, boxing, judo, jujitsu, karate, kick boxing, MMA, real aikido, savate boxing); cycling, 0.3%; dance, 3.5% (ballet, dance, folklore); equestrian sports, 0.05%; gymnastics, 2.1% (rhythmic gymnastics, artistic gymnastics); ice sports, 0.3% (ice hockey, figure skating); nautical sports, 0.4% (rowing, kayak); racquet sports, 0.5% (table tennis, tennis, badminton); small ball sports, 0.1% (cricket, bowl); team sports, 73.2% (volleyball, handball, football, basketball, American football, rugby); water sports, 8.2% (swimming, diving, water polo); and yoga, 0.05%. Due to the wide variety of sports, we have categorized them as follows: collective or individual; contact or non-contact.

As the Serbian education system in schools primarily teaches children between 11 and 18 years about aggression and violence (such as peer violence) constructs in the subjects of Civic Education, Psychology, and Sociology, the research assistants explained to athletes, their coaches, and their parents (guardians) the necessary terminologies for understanding the questionnaire at the beginning of the response process, such as the difference between aggression and violent behaviors. For clarity, the questions primarily focused on children’s perceptions and manifestation of violence rather than aggression to avoid any misunderstanding.

The sample in this study was divided into several categories based on the athletes’ ages (younger athletes (11–14 years of age) and older athletes (15–18 years of age)); training year (1–5 years of training and 5 or more years of training); and type of sport (individual and collective; contact and non-co ntact).

### 2.2. Procedure

#### Questionnaires

The data were collected via a questionnaire, which was included in other studies as well [33,34,35]. The research team designed a questionnaire focusing on the topic of aggression and interpersonal violence manifestation in their various forms and associated factors among athletes in youth sports. A preliminary study was conducted before the questionnaire was used for the first time, with *n* = 60 subjects. The questionnaire of the pilot study produced favorable metrics. It demonstrated satisfactory reliability, as the Cronbach alpha coefficient for internal consistency (α) was between 0.81 and 0.93. It also demonstrated validity, as the linear correlation analysis coefficient (r) was between 0.74 and 0.80. We have, therefore, included this questionnaire in our additional studies on this topic [33,34,35].

The questions were designed to investigate whether the respondents noticed and were aware of aggressive behavior or IV. The questionnaire is described in Table 1. It included 16 questions, each with one to twelve items, and it was divided into two segments. The first covered the socio-demographic characteristics of the athletes. The second segment included questions addressing peer violence. The answers were scored on a five-point Likert scale and ranged from completely disagreeing (one point) to completely agreeing (five points).

The athletes and their parents (guardians) read and gave informed consent for participation in this research. They were assured of anonymity and that their data would be kept confidential and used only for research purposes. Participation in this study was voluntary. Guardians, parents, club representatives, and athletes were informed about the objectives of this study. The questionnaire required a time of approximately 30 min to complete, including all necessary instructions, terminology explanations, and support provided by the research assistants and coaches. Data collection occurred at the beginning of training sessions at the sports club.

The aggression and IV manifestations and the influence of the athletes’ age, sports experience (training years), and type of sport were analyzed. The participants’ demographic characteristics were described using descriptive statistics. The differences were examined with the Chi-square and Mann–Whitney U tests in IBM SPSS version 24.0, with a significance level of *p* < 0.05.

## 3. Results

### 3.1. Demographic Characteristics for the Sample

The sample included *n* = 2091 participants, with 1363 boys and 874 girls, aged between 11 and 18. The majority (*n* = 1363) were categorized as younger athletes attending primary school (11–14 years of age), while *n* = 783 were classified as older athletes attending high school (15–18 years). *n* = 484 of them were living in villages or suburbs, while *n* = 1727 of them were living in a town or city. The participants’ sports experience was described based on their years of training: less than five years (*n* = 1226) or more than five years (*n* = 762). The classification of sports was initially associated with the nature of their competitors. Among the athletes, *n* = 473 trained in individual sports and *n* = 1747 in collective sports. Furthermore, categorization was performed according to the degree of physical interaction between athletes: *n* = 1516 were involved in contact sports and *n* = 704 in non-contact sports.

### 3.2. Aggression and Interpersonal Violence Occurrence

The results in Table 2 show that there are significant differences between the athlete group (based on various factors) in the manifestation of peer violence. The occurrence of violence is more frequent among older athletes (15–18 years of age) (older_Yes_ = 39.3%, younger_Yes_ = 28.0%, χ^2^ = 28.41, *p* < 0.01) and athletes from collective (collective_Yes_ = 35.6%, individual_Yes_ = 18.8%, χ^2^ = 48.13, *p* < 0.01) and contact (contact_Yes_ = 37.6%, non-contact_Yes_ = 20.2%, χ^2^ = 65.71, *p* < 0.01) sports.

### 3.3. Different Types of Aggression and Interpersonal Violence

Based on the results from Table 3, older athletes (15–18 years of age) (M_older_ = 1099.79, M_younger_ = 1007.43, U = 459,811.00, *p* < 0.01) and athletes from collective (M_collective_ = 1100.58, M_individual_ = 971.12, U = 343,224.50, *p* < 0.01) and contact sports (M_contact_ = 1104.83, M_non-contact_ = 1003.94, U = 453,380.50, *p* < 0.01) are more likely to use physical violence.

In addition, older athletes (15–18 years of age) (M_older_ = 1139.16, M_younger_ = 994.08, U = 439,444.00, *p* < 0.01) and athletes with five or more years of training (M_fiveormore_ = 1002.47, M_onetofive_ = 945.44, U = 417,404.50, *p* < 0.05) and from collective (M_collective_ = 1096.57, M_individual_ = 1015.07, U = 363,661.50, *p* < 0.01) and non-contact (M_non-contact_ = 1148.37, M_contact_ = 1046.58, U = 457,289.00, *p* < 0.01) sports demonstrate a higher likelihood for psychological violence.

## 4. Discussion

Many studies have investigated the association between participation in sports and aggressive and violent behavior [1,12,28]. In sports, these destructive behaviors can occur at all levels, from elite to recreational sports [10]. These behaviors can manifest in various ways, from bullying and teasing to hitting or kicking. In addition, they can also be linked to different causes and factors, such as the level and type of organized sports. The aim of the present study was to investigate different aspects (or indicators) of aggression and IV manifestation (physical, psychological, and sexual) and factors that may contribute to it. The factors included were the ages of the athletes (younger athletes (11–14 years of age) and older athletes (15–18 years of age)), sports experience (1–5 years of training and 5 or more years of training), and the type of sport (individual and collective; contact and non-contact). The results of this study show that aggression and IV occur in the youth sports context in Vojvodina, Serbia. These phenomena are not unknown but, in recent years, have received unprecedented attention [28]. These behaviors are also present in other countries, as 4000 adults from the Netherlands and Belgium reported retrospective experiences of interpersonal violence [21]. In addition, our results have shown that the athletes’ age and the type of sport (collective and contact sports) in which they participated are associated with aggression and IV manifestation in sports contexts. It is possible that differences in setting and sport type may contribute to aggression and IV in the sports domain [1].

First, we explored the manifestation of aggression and IV manifestation based on the athletes’ ages. The occurrence of violence, including both psychological and physical types, is more frequent among older athletes. These results are in line with an earlier finding that older adolescents demonstrated a greater tendency for aggressive behavior [26,36]. Likewise, athletes who were 17–20 years old demonstrated higher aggression scores than peers 26 years or older [37]. It is believed that adolescence is one of the most vulnerable phases for violence [28]. On the contrary, Ivanskiene et al. found no meaningful differences between athletes of different age categories [38].

Training and being in a sports context for a longer period might increase the possibility of encountering IV [8]. In the following analysis, we included years of training as a factor. We found out that based on the years of training, there were differences in psychological violence between children and youth. Athletes who have trained for five or more years show a higher tendency for this type of destructive behavior. However, Mazzanti et al. did not find significant differences between beginners and experts (who trained for more than 4 years) [1,39].

Interpreting the results from our comparison of athletes in contact and non-contact sports, we found that those who play contact sports tend to demonstrate a higher frequency of aggression and violence. Additionally, among the contact sports, physical violence was higher. On the other hand, psychological violence was more prevalent in non-contact sports. These results align with the research of Kreager, who reported that high-contact sports (e.g., football, wrestling) led to increased violent behavior, whereas non-contact sports did not [32]. Studies such as [10] confirm that aggression may depend on the contact type of the sport. Similarly, Sofia and Cruz indicated that athletes from sports with higher levels of physical contact are more likely to be aggressive [12]. These high-contact sports tend to involve intense competition, which can often lead to impulsive behavior. A comparison of aggression between youth who play sports and those who do not showed that athletes from non-contact sports exhibited lower levels of verbal aggression than non-athletes or athletes from combat and contact sports [40]. It is also worth noticing that combat sports, by their nature, allow some physical attacks that can be associated with aggression. However, these attacks must be aligned with the regulations and rules of that sport [41]. For other types of aggressive behavior, we find similar results. Compared to other sports, wrestlers stand out due to a higher number of bullying victims and perpetrators [42].

When comparing athletes by other categories of sport type (individual and team/collective), we found that violence was more common in collective sports. Both physical and psychological violence were more likely in collective sports. Team sports could, therefore, be associated with the occurrence of aggression and IV [1], but sometimes, the opposite is also true [39]. Weinhardt and Fominiene showed that almost one-third of athletes in team sports have been identified as victims of bullying [23]. The results of our study, as described, highlight the need for targeted educational programs aimed at reducing aggression and interpersonal violence in youth sports. Given that physical violence is more prevalent among older athletes in contact and team sports, while psychological violence is more common among athletes with five or more years of experience, sports organizations and coaches need to develop tailored prevention strategies. Previous studies [22,43] suggest that structured anti-bullying programs, training coaches in conflict resolution, and fostering a positive team culture can effectively reduce violent behavior. In addition, integrating peer mentoring programs and psychological support services into sports environments may help mitigate the long-term consequences of exposure to violence in sports. Future research should explore how these interventions affect athletes’ behavior over time, as well as the role of sport-specific factors in shaping aggressive tendencies.

Safeguarding athletes from aggression and IV is important [44]. Consequently, it is necessary to identify settings and factors that can have an influence on aggressive behavior and IV to obtain empirical data that can serve as a basis for further theoretical guidance to reduce these destructive behaviors [45]. This is important, as we know that athletes experience all types of IV (psychological, physical, and sexual) in sports [8,31].

This study has several limitations and potential areas for improvement. First, the sample does not represent the population of Serbian youth athletes, as it was conducted in one part of society (in the Autonomous Province of Vojvodina), although this part represents an autonomous and relatively independent social and administrative unit. Second, sports categories were not equally included in this study. Third, the implementation of COVID-19 pandemic emergency measures brought further challenges to the research process. Future research should include the following recommendations: It should be extended to other parts of Serbia, not only the northern part, to better represent the situation in this country. In addition, an equal representation of the different sport types would better describe the context of the manifestations of these destructive behaviors and their associated factors. Lastly, a more comprehensive sport-type division could analyze the context more accurately.

## 5. Conclusions

This study provides several insights into the factors influencing the manifestation of aggression and IV in youth sports. By examining athletes’ age, training experience, and sport type, we identified a few patterns that contribute to these behaviors. Our results highlighted that athletes’ age, years of training, and the type of sport they participate in can influence the likelihood of physical or psychological violent behaviors. Specifically, physical violence tends to be more common among older athletes in collective and contact sports, while psychological violence is more prevalent among older athletes who have trained for five or more years and participate in collective and non-contact sports. By addressing and identifying some of the factors that correlate with the manifestation of aggression and IV, stakeholders can develop strategies to reduce these destructive behaviors in youth sports environments.

## Figures and Tables

**Table 1:** Questionnaire used for aggression and IV manifestation.

Questionnaire Structure	
Socio-demographic Characteristics of the Athletes	
gender age the place of residence the number of people and children in the family parents’ education level school attendance type of sport they trained previous sports experience	
Aggression and IV manifestation and types	
occurrence	(yes, no)
frequency	(from never to very often)
timing	(before, during, or after training, competitions, trips, and sports camps)
location (place)	(sports hall, dressing room, training, bathroom areas)
acts	(taunting, insults, threats, messages, recordings, pushing, choking, showing and touching inappropriate parts)
forms of violence - that can occur in theory based on their opinions (theory) - forms that most frequently occur (in practice) to cover all aspects of the experience of these destructive behaviors	physical (e.g., hitting, biting, pushing) psychological (e.g., insulting, mocking, gossiping) sexual (inappropriate touching, showing of genitals, humiliation related to gender issues)
Understanding the dynamics	
the characteristics of the victims of violence and those who perpetrate it the reasons and consequences of violence the ways that athletes, parents, coaches, and other actors can react against these destructive behaviors	

**Table 2:** Results of Chi-squared test.

Variables	Is There Violence in the Sport You Play? (%)		How Often Does Violence in Your Sport Occur Among Children? (%)				
	Yes	No	Very Often	Quite Often	Sometimes	Rarely	Almost Never
Younger athletes (11–14 years of age)	28.0	72.0	2.6	1.5	15.1	26.5	54.2
Older athletes (15–18 years of age)	39.3	60.7	1.2	4.7	20.9	30.1	43.1
	χ^2^ = 28.41 **		χ^2^ = 46.05 **				
1–5 years of training	32.2	67.8	2.6	2.4	17.0	27.8	50.1
5 or more years of training	34.6	65.4	1.5	2.6	18.9	29.0	48.0
	χ^2^ = 1.17		χ^2^ = 4.16				
Individual sport	18.8	81.2	1.3	2.4	9.4	21.3	65.6
Collective sport	35.6	64.4	2.3	2.8	19.1	29.3	46.4
	χ^2^ = 48.13 **		χ^2^ = 56.07 **				
Contact	37.6	62.4	2.8	3.1	20.1	30.0	44.0
Non-contact	20.2	79.8	0.6	1.8	10.4	22.6	64.6
	χ^2^ = 65.71 **		χ^2^ = 88.15 **				

Legend: **—*p* < 0.01.

**Table 3:** Results of Mann–Whitney U test among variables in the forms of violence.

Variables	Forms of Violence that Occurred Most Frequently (Mean Rank)		
	Physical Violence	Psychological Violence	Sexual Violence
Younger athletes (11–14 years of age)	1007.43	994.08	1021.31
Older athletes (15–18 years of age)	1099.79 **	1139.16 **	1062.34
1–5 years of training	966.34	945.44	951.83
5 or more years of training	952.58	1002.47 *	961.25
Individual sport	971.12	1015.07	1054.88
Collective sport	1100.58 **	1096.57 **	1069.09
Contact	1104.83 **	1046.58	1079.59
Non-contact	1003.94	1148.37 **	1037.18

Legend: *—*p* < 0.05, **—*p* < 0.01.

## Data Availability

The raw data supporting the conclusions of this article will be made available by the corresponding author without undue reservation.

## Abbreviations

The following abbreviations are used in this manuscript:

IV	Interpersonal violence
